# The *is* and *ought* of the Ethics of Neuroenhancement: Mind the Gap

**DOI:** 10.3389/fpsyg.2015.01998

**Published:** 2016-01-08

**Authors:** Cynthia Forlini, Wayne Hall

**Affiliations:** ^1^The University of Queensland Centre for Clinical Research, The University of Queensland, BrisbaneQLD, Australia; ^2^The University of Queensland Centre for Youth Substance Abuse Research, The University of Queensland, BrisbaneQLD, Australia; ^3^National Addiction Centre, King’s College LondonLondon, UK

**Keywords:** neuroenhancement, neuroethics, normative ethics, empirical ethics, values, stakeholders, behavior

## Abstract

Ethical perspectives on the use of stimulants to enhance human cognitive performance (neuroenhancement) are polarized between conservative and liberal theories offering opposing advice on whether individuals have a right to use neuroenhancers and what the social outcomes of neuroenhancement might be. Meanwhile, empirical evidence shows modest prevalence and guarded public attitudes toward the neuroenhancement use of stimulants. In this *Perspective*, we argue that the dissonance between the prescriptions of ethical theories (what ought to be) and empirical evidence (what is) has impaired our understanding of neuroenhancement practices. This dissonance is a result of three common errors in research on the ethics of neuroenhancement: (1) expecting that public perspectives will conform to a prescriptive ethical framework; (2) ignoring the socio-economic infrastructures that influence individuals’ decisions on whether or not to use neuroenhancement; and (3) overlooking conflicts between fundamental ethical values namely, safety of neuroenhancement and autonomy. We argue that in order to understand neuroenhancement practices it is essential to recognize which values affect individual decisions to use or refuse to use neuroenhancement. Future research on the ethics of neuroenhancement should assess the morally significant values for stakeholders. This will fill the gap between what ought to be done and what is done with an improved understanding of what *can* be done within a particular context. Clarifying conflicts between competing moral values is critical in conducting research on the efficacy of substances putatively used for neuroenhancement and also on neuroenhancement practices within academic, professional and social environments.

## Background

Ethical perspectives on the use of substances to enhance human cognitive performance (neuroenhancement) have become polarized between conservative and liberal normative theories (Racine, 2010; Forlini and Racine, 2013). These theories stem from respective political stances on technology and human enhancement that fundamentally disagree about whether individuals have a right to use neuroenhancers and about the potential social outcomes of neuroenhancement (Hughes, 2009; Reiner, 2013).

The *liberal* or *meliorist* approach maintains that “[h]uman history — or at least human progress — is in great part the story of enhancement” (Buchanan, 2010) as reflected in the development of tools, technology, and organized societies. Evolution, for its part, might be considered as the “original” process that enhanced human capacities and characteristics. From this standpoint enhancement should continue to be pursued because it promises to reduce suffering and improve the quality of human life (Caplan, 2003; Savulescu, 2006; Harris, 2007; Bostrom and Sandberg, 2009; Buchanan, 2010). At the extreme of this liberal perspective is *transhumanism*, a movement that embraces science and technology in the hope of becoming “post-human, beings with vastly greater capacities than present human beings have” (Bostrom, 2003).

The *conservative* or *anti-meliorist* standpoint strives to preserve “human nature”. From this position, enhancement poses a risk to human existence because it may produce undesirable physical and social changes in human beings (Fukuyama, 2002; President’s Council on Bioethics, 2003; Sandel, 2004). Evolution, conservatives argue, should not to be meddled with. The risk is that in “enjoying the benefits of biotechnology, we will need to hold fast to an account of the human being, seen not in material or mechanistic or medical terms but in psychic and moral and spiritual ones” (President’s Council on Bioethics, 2003). Biotechnology erodes the building blocks of the “human dignity” as exemplified by the discipline and effort that are required to attain excellence and that promote human flourishing and our identity (Kass, 2003).

There are many more nuanced positions between these conservative and liberal views (Hughes, 2009; Reiner, 2013). However, these opposing points of view represent extremes in the broader “culture wars” that underlie bioethics debates in the USA about stem cell research and end-of-life care (Callahan, 2005; Hughes, 2009; Racine, 2010). The “culture wars” reflect “radical moral-political divisions in the public domain” (Racine, 2010) that mirror disagreements between conservative and liberal moral and political positions. The fundamental differences between conservative and liberal approaches to enhancement make it difficult to come to a shared understanding of how to proceed at the institutional and community levels. Some authors have declared an ethical stalemate because they believe that the conservative and liberal positions can never be reconciled and so cannot produce ethical advice for stakeholders (e.g., students, health professionals, policy makers, academic institutions, members of the general public) (Roache and Clarke, 2009; Banja, 2011).

Despite their differences, these two poles in the neuroenhancement ethics debate both unwittingly and uncritically promote the “myth of cognitive enhancement” (Zohny, 2015). That is, they both assume that putatively neuroenhancing substances do in fact enhance and that their use is widespread and increasing. Neither of these assumptions is well supported by empirical evidence (Lucke et al., 2011).

First, most of the prescription medications labeled as neuroenhancers (i.e., prescription stimulants such as Ritalin, Adderall and Modafinil) have little, if any, enhancing effect in healthy individuals (Repantis et al., 2010a,b). A recent systematic review reported that modafinil provides some benefit in complex tasks but criticized the studies for their lack of sensitivity, reproducibility and ecological validity (Battleday and Brem, 2015).

Second, the empirical survey evidence finds a very modest prevalence of neuroenhancement use of stimulants, even in academic environments, an alleged hotspot of use (Smith and Farah, 2011). Public attitudes toward their use are guarded, but not entirely conservative, reflecting a politically moderate stance that is sensitive to salient ethical issues (Fitz et al., 2014). Public attitudes also vary according context and experience with neuroenhancement (Schelle et al., 2014). These survey data do not support the claims of widespread and increasing neuroenhancement use often made by proponents of the two dominant normative approaches.

Why do stakeholder views and actions differ from what is widely assumed by the two ethical perspectives? How should we marry normative theories (what ought to be) with empirical evidence (what is) in this bioethics debate? As several authors have argued, it is important to bring the two together because it is “not only sufficient for an ethicist to discuss the moral rightness or wrongness of a certain practice on a theoretical level, but also to think about the conditions under which a norm can be effective in society” (Birnbacher, 1999) (de Vries and Gordijn, 2009; Salloch et al., 2012). In this *Perspective*, we argue that the dissonance between the opposing normative theories and empirical data on stakeholder attitudes has impaired our understanding of neuroenhancement practices. We describe key features of this dissonance and outline an approach to research on the ethics of neuroenhancement that may help to bridge the gap between normative and empirical perspectives.

## Three Common Errors in Research on the Ethics of Neuroenhancement

Empirical reality often limits human agency in ways that conflict with normative or theoretical views (Potter, 1971; Hurst, 2010). In the context of neuroenhancement, there are at least three instances in which real life situations have contributed to errors in ethical reflection.

### Expecting that Public Perspectives will Conform to a Prescriptive Ethical Framework

Studies that elicit the perspectives of stakeholders on neuroenhancement often identify the same issues discussed in academic discourse on the topic (Schelle et al., 2014). However, there are two important differences between stakeholder perspectives and formal normative reflections. First, stakeholder studies report the coexistence within individuals of conflicting ethical perspectives (i.e., ambivalence) on neuroenhancement and its acceptability in medical and academic environments (Banjo et al., 2010; Hotze et al., 2011; Forlini and Racine, 2012b). This ambivalence is often evident in students’ reactions to analogies between neuroenhancement use of caffeine and sports doping, which can be taken as representing liberal and conservative perspectives, respectively. Two studies have shown that students analogise neuroenhancement and sports doping in competition but differentiate the two on the basis of the magnitude of steroid effects compared to the effects of caffeine or prescription stimulants (Forlini and Racine, 2012b; Bell et al., 2013). In another study, 56% of a sample of German university students saw no *moral* difference between neuroenhancement and the use of caffeinated substances. However, 44% also said that prescription stimulants and caffeine differed in their side effects, medical risks and legal consequences (e.g., in using a medical prescribed substance illegally). Just under half (46%) of these students either *did not* or *could not* differentiate the two types of substances. These students were uncertain about the most appropriate policy framework (permissive or restrictive).

Second, the ethical acceptability of neuroenhancement seems to be a matter of degree for many stakeholders. They often express reservations about its acceptability or specify conditions under which it would be ethically acceptable. For example, neuroenhancement could be acceptable to some if its use was controlled and moderate (Forlini and Racine, 2009; Bell et al., 2013). The use of neuroenhancing substances was found to be more acceptable when used to: *enhance to the norm* (Cabrera et al., 2015), *enable* the true self (Riis et al., 2008), *normalize* the performance of underperforming colleagues (Sabini and Monterosso, 2005) or *restore* cognitive function caused by normal age-associated memory impairment (Banjo et al., 2010) than when used to *enhance above the norm*. The more acceptable conditions of use were thought to reflect degree of medical necessity, a key factor for many in distinguishing between enhancement and treatment (Cabrera et al., 2015) as well as a concern for fairness and equality of opportunity (Sabini and Monterosso, 2005). Acceptability also varied with whether the target for enhancement was seen as connected with the authenticity of the individual (Riis et al., 2008; Berg et al., 2009). Stakeholders seemed less willing to accept the neuroenhancement of mood, emotions, and memory because they were seen to be more closely associated with self-identity than aspects such as concentration or alertness. These empirical findings on the degrees of acceptability of neuroenhancement may not fit neatly into the principled approach of normative ethics. They also challenge the myth of widespread stakeholder interest in neuroenhancement (Farah et al., 2004; Greely et al., 2008) because stakeholder perspectives are not as resoundingly liberal as often assumed by proponents of enhancement.

### Ignoring the Socio-Economic Infrastructures That Influence Decisions

Empirical ethics research not only studies the normative values held by stakeholders; it also describes their actions and behaviors in situations that call for a moral choice. Empirical inquiry has revealed that some stakeholders feel under pressure to perform and believe that they have “no choice” except to use neuroenhancers, despite also believing that this choice should be an individual or personal matter (Forlini and Racine, 2009). Stakeholders are also more willing to use neuroenhancers if they believe that their peers are doing so in order to avoid being at a social disadvantage (Franke et al., 2012a; Sattler et al., 2013). Other studies have found more modest (Fitz et al., 2014) and sometimes opposite effects of peer pressure (Forlini et al., 2015). These differences may reflect cultural or contextual differences in stakeholder perspectives that merit further investigation.

Equality of access was another major ethical concern for stakeholders. The majority agreed that if neuroenhancement was allowed then it should be available to everyone but very few thought that the cost of neuroenhancers should be covered by health insurance (Bergstrom and Lynoe, 2008; Hotze et al., 2011; Forlini and Racine, 2012a). The roles of peer pressure and equality of access show that professed ethical values do not always translate into behavior and that socioeconomic factors may influence decisions to use or not to use neuroenhancement as much as ethical values.

Bioethicists often assume “an implausible degree of rationality”, individual freedom and consistency in human decisions, motivations, and actions (Solomon, 2005). An individual’s decision to use neuroenhancement is multi-faceted and ethical norms may only comprise one factor. There may also be inconsistencies between personal values and behavior because when “faced with an ethical dilemma, people do not always think about what they ought to do in isolation from what they are currently doing” (Ives and Draper, 2009). Fear of being at a disadvantage, or worries about scarce health resources, may influence attitudes toward enhancement more than values like personal choice or equality of opportunity. Schelle et al. (2014) postulate that stakeholders, especially neuroenhancement users, can experience cognitive dissonance, which is “the discomfort experienced when one’s actions don’t reflect one’s beliefs.” This dissonance will persist until values catch up with the demands of the socioeconomic context in which they are meant to govern behavior.

### Overlooking Incompatibilities Between Fundamental Values

Empirical data shows that stakeholders have difficulty balancing safety and autonomy. On the one hand, many stakeholders believe that using neuroenhancement is a personal choice for which individuals must take responsibility (Forlini and Racine, 2009; Franke et al., 2012a; Bell et al., 2013). Part of this responsibility is to make decisions that may affect one’s health (Forlini and Racine, 2009; Banjo et al., 2010) and to evaluate the risks of doing so. Neuroenhancers are prepared to tolerate mild to moderate adverse side effects but are deterred by the prospects of long-term and serious side effects (Franke et al., 2012b; Sattler et al., 2013). These beliefs reflect the liberal perspective on neuroenhancement which champions the autonomy of individuals and their right to incur whatever risks that they are comfortable with incurring (Sententia, 2004). On the other hand, for many stakeholders safety is paramount. Even when presented with a hypothetically safe cognitive enhancer in a study vignette, Banjo et al. (2010) found that, “physicians mistrust safety claims regarding pharmaceuticals.” For these physicians, safety concerns “were not offset by the benefit afforded the individual” (Banjo et al., 2010). Similarly, Hotze et al. (2011) reported that a hypothetically safe neuroenhancer was seen as unacceptable because they were regarded as being “too risky” (for reasons unspecified in the survey) or likely to cause negative behaviors (e.g., making a soldier more aggressive). These perspectives are consistent with the conservative stance that the uncertain safety of neuroenhancement trumps individual autonomy (Heinz et al., 2012).

Stakeholders’ perspectives on the values of safety and autonomy are well defined but the challenge is to balance the two in ways that place socially agreed limits on acceptable forms of neuroenhancement. The use of an enhancer that was unsafe and used under coercion would be unacceptable, regardless of political stance. Given this, it is may be useful to explore stakeholder trade-offs between these two values under scenarios that vary by *degrees in* safety and autonomy. This would illuminate the level of risk that was seen as appropriate for an individual to take in pursuit of neuroenhancement and views on the extent to which individuals should be prevented from taking this risk to protect them from harm.

## Bridging *is* and *Ought* with “Can”

Empirical data can help bioethicists to understand the values of stakeholders so that theory and policies can be made more relevant and effective (Ives and Draper, 2009). If principles “are too abstract or practically not feasible” then normative ethics fails to guide action (de Vries and Gordijn, 2009). Until normative-empirical tensions are unwound it would be difficult to carry out a constructive discourse that informs policy. As more empirical data on stakeholder perspectives about the ethics of neuroenhancement emerge, normative bioethicists may have to revisit their discussions of ethical principles for two reasons. First, this process may create a negotiated space between what people “ought” to do and what they actually “can” do within existing socio-economic frameworks. Discovering what can be done will require normative and empirical research to test the degrees of public acceptability. This may identify an “ethical tipping point” in a debate still plagued with ambivalence about many of the salient issues. Second, empirical data may uncover unethical behavior that does not respect traditional social values. If, for example, we discovered that certain professional environments obliged employees to take cognitive enhancers in order to be more productive most would object on the grounds that this practice was coercive, regardless of whether or not the enhancer was safe.

Normative ethics might also have the task of reinforcing values and promoting ethical behavior. Drawing attention to and working through tensions between normative principles and behavior may help to refine the most significant ethical values for stakeholders. This exercise is sorely needed to, first, distance research on the ethics of neuroenhancement from the culture wars and an uncritical acceptance of the myths of cognitive enhancement, and second, to increase social dialogue and deliberation on the acceptability of neuroenhancement and thereby reinvigorate the mandate of bioethics to enrich societal perspectives by closely examining contentious issues.

## Conclusion

Future research on the ethics of neuroenhancement should assess the values that are most morally significant to the public in order to better understand how the public approaches neuroenhancement. We need to fill the gap between what ought to be done and what is done by a better understanding of what *can* be done in a particular social context. In doing so, we should avoid assuming that principles and practices, concepts, and experience will not change. As Solomon (2005) comments, practice “guidelines persist, taking a life if their own, while the conditions that motivated them change.” It is the responsibility of bioethicists to “formulate and reformulate our ethical theories” (Frith, 2012) to keep the reflections relevant to public policy debates.

## Author Contributions

CF drafted, revised, and finalized the manuscript. WH refined content and revised the manuscript.

## Conflict of Interest Statement

The authors declare that the research was conducted in the absence of any commercial or financial relationships that could be construed as a potential conflict of interest.

## References

[B1] Banja J. (2011). Virtue essentialism, prototypes, and the moral conservative opposition to enhancement: a neuroethical critique. *AJOB Neurosci.* 2 31–38. 10.1080/21507740.2011.556918

[B2] Banjo O. C. Nadler R. Reiner P. B. (2010). Physician attitudes towards pharmacological cognitive enhancement: safety concerns are paramount. *PLoS ONE* 5:e14322 10.1371/journal.pone.0014322 PMC3001858 21179461

[B3] Battleday R. M. Brem A. K. (2015). Modafinil for cognitive neuroenhancement in healthy non-sleep-deprived subjects: a systematic review. *Eur. Neuropsychopharmacol.* 25 1865–1881. 10.1016/j.euroneuro.2015.07.028 26381811

[B4] Bell S. Partridge B. Lucke J. Hall W. (2013). Australian university students’ attitudes towards the acceptability and regulation of pharmaceuticals to improve academic performance. *Neuroethics* 6 197–205. 10.1007/s12152-012-9153-9

[B5] Berg J. W. Mehlman M. J. Rubin D. B. Kodish E. (2009). Making all the children above average: ethical and regulatory concerns for pediatricians in pediatric enhancement research. *Clin. Pediatr.* 48 472–480. 10.1177/0009922808330457 19164131

[B6] Bergstrom L. S. Lynoe N. (2008). Enhancing concentration, mood and memory in healthy individuals: an empirical study of attitudes among general practitioners and the general population. *Scand. J. Public Health* 36 532–537. 10.1177/1403494807087558 18635734

[B7] Birnbacher D. (1999). Ethics and social science: which kind of cooperation? Ethical theor. *Moral Pract.* 2 319–336. 10.1023/A:1026409110084 15015521

[B8] Bostrom N. (2003). Human genetic enhancements: a transhumanist perspective. *J. Value Inq.* 37 493–506. 10.1023/B:INQU.0000019037.67783.d5 17340768

[B9] Bostrom N. Sandberg A. (2009). Cognitive enhancement: methods, ethics, regulatory challenges. *Sci. Eng. Ethics* 15 311–341. 10.1007/s11948-009-9142-5 19543814

[B10] Buchanan A. (2010). *Better Than Human: The Promise and Perils of Enhancing Ourselves.* Oxford: Oxford University Press.

[B11] Cabrera L. Fitz N. S. Reiner P. B. (2015). Empirical support for the moral salience of the therapy-enhancement distinction in the debate over cognitive, affective and social enhancement. *Neuroethics* 8 243–256. 10.1007/s12152-014-9223-2

[B12] Callahan D. (2005). Bioethics and the culture wars. *Camb. Q. Healthc. Ethics* 14 424–431. 16250496 10.1017/s0963180105050577

[B13] Caplan A. L. (2003). Is better best? A noted ethicist argues in favor of brain enhancement. *Sci. Am.* 289 104–105. 10.1038/scientificamerican0903-104 12951834

[B14] de Vries R. Gordijn B. (2009). Empirical ethics and its alleged meta-ethical fallacies. *Bioethics* 23 193–201. 10.1111/j.1467-8519.2009.01710.x 19338520

[B15] Farah M. J. Illes J. Cook-Deegan R. Gardner H. Kandel E. King P. (2004). Neurocognitive enhancement: what can we do and what should we do? *Nat. Rev. Neurosci.* 5 421–425. 10.1038/nrn1390 15100724

[B16] Fitz N. S. Nadler R. Manogaran P. Chong E. W. J. Reiner P. B. (2014). Public attitudes toward cognitive enhancement. *Neuroethics* 7 173–188. 10.1007/s12152-013-9190-z

[B17] Forlini C. Racine E. (2009). Autonomy and coercion in academic “cognitive enhancement” using methylphenidate: perspectives of a pragmatic study of key stakeholders. *Neuroethics* 2 163–177. 10.1007/s12152-009-9043-y

[B18] Forlini C. Racine E. (2012a). Added value(s) to the cognitive enhancement debate: are we sidestepping values in academic discourse and professional policies? *AJOB Primary Res.* 3 33–47. 10.1080/21507716.2011.645116

[B19] Forlini C. Racine E. (2012b). Stakeholder perspectives and reactions to “academic” cognitive enhancement: unsuspected meaning of ambivalence and analogies. *Public Underst. Sci.* 21 606–625. 10.1177/0963662510385062 23823168

[B20] Forlini C. Racine E. (2013). “Does the cognitive enhancement debate call for a renewal of the deliberative role of bioethics?,” in *Cognitive Enhancement: An Interdisciplinary Perspective*, eds Hildt E. Franke A. (New York, NY: Springer), 173–186.

[B21] Forlini C. Schildmann J. Roser P. Beranek R. Vollmann J. (2015). Knowledge, experiences and views of German university students toward neuroenhancement: an empirical-ethical analysis. *Neuroethics* 8 83–92. 10.1007/s12152-014-9218-z

[B22] Franke A. G. Bonertz C. Christmann M. Engeser S. Lieb K. (2012a). Attitudes toward cognitive enhancement in users and nonusers of stimulants for cognitive enhancement: a pilot study. *AJOB Primary Res.* 3 48–57. 10.1080/21507716.2011.608411

[B23] Franke A. G. Lieb K. Hildt E. (2012b). What users think about the differences between caffeine and illicit/prescription stimulants for cognitive enhancement. *PLoS ONE* 7:e40047 10.1371/journal.pone.0040047 PMC3386931 22768218

[B24] Frith L. (2012). Symbiotic empirical ethics: a practical methodology. *Bioethics* 26 198–206. 10.1111/j.1467-8519.2010.01843.x 21039690

[B25] Fukuyama F. (2002). *Our Posthuman Future.* New York, NY: Picador.

[B26] Greely H. Sahakian B. Harris J. Kessler R. C. Gazzaniga M. Campbell P. (2008). Towards responsible use of cognitive-enhancing drugs by the healthy. *Nature* 456 702–705. 10.1038/456702a 19060880

[B27] Harris J. (2007). *Enhancing Evolution: The Ethical Case for Making People Better.* Princeton: Princeton University Press.

[B28] Heinz A. Kipke R. Heimann H. Wiesing U. (2012). Cognitive neuroenhancement: false assumptions in the ethical debate. *J. Med. Ethics* 38 372–375. 10.1136/medethics-2011-100041 22228818

[B29] Hotze T. Shaw K. Anderson E. Wynia M. (2011). Doctor, would you prescribe a pill to help me…?” A national survey of physicians on using medicine for human enhancement. *Am. J. Bioeth.* 11 3–13. 10.1080/15265161.2011.534957 21240795

[B30] Hughes J. (2009). “*Technoprogressive Biopolitics and Human Enhancement,” in Progress in Bioethics.* eds Moreno J. Berger S. (Cambridge, MA: MIT Press), 163–188.

[B31] Hurst S. (2010). What ‘empirical turn in bioethics?’ *Bioethics* 24 439–444. 10.1111/j.1467-8519.2009.01720.x 19438442

[B32] Ives J. Draper H. (2009). Appropriate methodologies for empirical bioethics: it’s all relative. *Bioethics* 23 249–258. 10.1111/j.1467-8519.2009.01715.x 19338525

[B33] Kass L. (2003). *Ageless Bodies, Happy Souls.* New York, NY: The New Atlantis Spring, 9–28. 15584192

[B34] Lucke J. Bell S. Partridge B. Hall W. D. (2011). Deflating the neuroenhancement bubble. *AJOB Neurosci.* 2 38–43. 10.1080/21507740.2011.611122

[B35] Potter V. R. (1971). *Bioethics: Bridge to the Future.* Englewood Cliffs, NJ: Prentice-Hall.

[B36] President’s Council on Bioethics (2003). *Beyond Therapy.* Washington, DC: President’s Council on Bioethics/Harper Collins, 328.

[B37] Racine E. (2010). *Pragmatic Neuroethics: Improving Treatment and Understanding of the Mind-Brain.* Cambridge, MA: MIT Press.

[B38] Reiner P. B. (2013). “The biopolitics of cognitive enhancement,” in *Cognitive Enhancement: An Interdisciplinary Perspective*, eds Hildt E. Franke A. (New York, NY: Springer), 189–200.

[B39] Repantis D. Laisney O. Heuser I. (2010a). Acetylcholinesterase inhibitors and memantine for neuroenhancement in healthy individuals: a systematic review. *Pharmacol. Res.* 61 473–481. 10.1016/j.phrs.2010.02.009 20193764

[B40] Repantis D. Schlattmann P. Laisney O. Heuser I. (2010b). Modafinil and methylphenidate for neuroenhancement in healthy individuals: a systematic review. *Pharmacol. Res.* 62 187–206. 10.1016/j.phrs.2010.04.002 20416377

[B41] Riis J. Simmons J. P. Goodwin G. P. (2008). Preferences for enhancement pharmaceuticals: the reluctance to enhance fundamental traits. *J. Consum. Res.* 35 495–508. 10.1086/588746

[B42] Roache R. Clarke S. (2009). Bioconservatism, bioliberalism, and the wisdom of reflecting on repugnance. *Monash Bioethics Rev.* 28 1–21. 10.1007/BF03351306 19839275

[B43] Sabini J. Monterosso J. (2005). Judgments of the fairness of using performance enhancing drugs. *Ethics Behav.* 15 81–94. 10.1207/s15327019eb1501_6 16127858

[B44] Salloch S. Schildmann J. Vollmann J. (2012). Empirical research in medical ethics: how conceptual accounts on normative-empirical collaboration may improve research practice. *BMC Med. Ethics* 13:5 10.1186/1472-6939-13-5 PMC3355047 22500496

[B45] Sandel M. J. (2004). The case against perfection: what’s wrong with designer children, bionic athletes, and genetic engineering. *Atl. Mon.* 292 50–54. 15468473

[B46] Sattler S. Forlini C. Racine E. Sauer C. (2013). Impact of contextual factors and substance characteristics on perspectives toward cognitive enhancement. *PLoS ONE* 8:e71452 10.1371/journal.pone.0071452 PMC3733969 23940757

[B47] Savulescu J. (2006). Justice, fairness, and enhancement. *Ann. N. Y. Acad. Sci.* 1093 321–338. 10.1196/annals.1382.021 17312266

[B48] Schelle K. J. Faulmuller N. Caviola L. Hewstone M. (2014). Attitudes toward pharmacological cognitive enhancement—a review. *Front. Syst. Neurosci.* 8:53 10.3389/fnsys.2014.00053 PMC4029025 24860438

[B49] Sententia W. (2004). Neuroethical considerations: cognitive liberty and converging technologies for improving human cognition. *Ann. N. Y. Acad. Sci.* 1013 221–228. 10.1196/annals.1305.014 15194617

[B50] Smith E. M. Farah M. J. (2011). Are prescription stimulants “smart pills?” The epidemiology and cognitive neuroscience of prescription stimulant use by normal healthy individuals. *Psychol. Bull.* 137 717–741. 10.1037/a0023825 21859174 PMC3591814

[B51] Solomon M. Z. (2005). Realizing bioethics’ goals in practice: ten ways “is” can help “ought.” *Hastings Cent. Rep.* 35 40–47. 10.2307/3528827 16225305

[B52] Zohny H. (2015). The myth of cognitive enhancement. *Neuroethics* 8 257–269. 10.1007/s12152-015-9232-9

